# Reduced Theta Sampling in Infants at Risk for Dyslexia across the Sensitive Period of Native Phoneme Learning

**DOI:** 10.3390/ijerph19031180

**Published:** 2022-01-21

**Authors:** Maria Mittag, Eric Larson, Samu Taulu, Maggie Clarke, Patricia K. Kuhl

**Affiliations:** 1 Institute for Learning & Brain Sciences, University of Washington, Seattle, WA 98195-7988, USA; larsoner@uw.edu (E.L.); staulu@uw.edu (S.T.); mdclarke@uw.edu (M.C.); 2 Department of Physics, University of Washington, Seattle, WA 98195-7988, USA

**Keywords:** dyslexia, infant, auditory, temporal sampling, MEG

## Abstract

Research on children and adults with developmental dyslexia—a specific difficulty in learning to read and spell—suggests that phonological deficits in dyslexia are linked to basic auditory deficits in temporal sampling. However, it remains undetermined whether such deficits are already present in infancy, especially during the sensitive period when the auditory system specializes in native phoneme perception. Because dyslexia is strongly hereditary, it is possible to examine infants for early predictors of the condition before detectable symptoms emerge. This study examines low-level auditory temporal sampling in infants at risk for dyslexia across the sensitive period of native phoneme learning. Using magnetoencephalography (MEG), we found deficient auditory sampling at theta in at-risk infants at both 6 and 12 months, indicating atypical auditory sampling at the syllabic rate in those infants across the sensitive period for native-language phoneme learning. This interpretation is supported by our additional finding that auditory sampling at theta predicted later vocabulary comprehension, nonlinguistic communication and the ability to combine words. Our results indicate a possible early marker of risk for dyslexia.

## 1. Introduction

Cortical oscillations are believed to play an important role in several aspects of human cognition, including attention, learning, object recognition, sensory feature binding, and emotional evaluation, and in context of the present work, speech perception and processing [1,2,3,4]. Information in speech unfolds over multiple temporal scales, and the brain processes amplitude modulations at different rates simultaneously as part of speech encoding [5]. In the auditory cortex, intrinsic oscillations can be detected at rest in discrete frequency ranges, predominantly at delta/theta (1–8 Hz), alpha (9–12 Hz), and low gamma (25–35 Hz) [6,7,8]. When the auditory cortex is stimulated by speech, these intrinsic oscillations give way to temporally structured activity that closely corresponds to the spectrotemporal structure of the speech envelope, with preferential tracking of speech modulations within delta (~2 Hz, prosodic rate), theta (4–8 Hz; syllabic rate) and gamma bands (>30 Hz; phonemic rate) [7,8].

Developmental dyslexia is defined as a specific learning difficulty in reading and spelling that cannot be attributed to poor cognitive and academic abilities or sensory impairments [9]. Children and adults with developmental dyslexia exhibit poor phonological processing across languages and orthographies, affecting the ability to recognize and manipulate the sound structure of words [10]. Recent research linked phonological deficits in dyslexia to atypical temporal coding of the speech signal by auditory cortical oscillatory networks that operate at different timescales [11,12]. Specifically, in children and adults with dyslexia, neural encoding of the speech signal was found to be impaired at delta/theta (1–8 Hz) and gamma (>30 Hz), both modulation frequency rates necessary for accurate speech perception [13,14,15,16,17,18,19,20,21,22,23,24,25,26].

The development of accurate auditory sampling in the infant brain has been implicated to contribute to later precise processing of spectrotemporal features of speech sounds, the generation of native phoneme representations, and later language and reading skills [11,27]. Synchronous bursts of activity between subgroups of neurons are already present at birth [28], which make it possible to examine oscillatory rhythms in the newborn brain. Researchers successfully recorded oscillations using different techniques such as electroencephalography (EEG) and hemodynamic near-infrared spectroscopy (NIRS) in typically developing (TD) infants from birth to 15 months [29,30,31,32,33,34,35,36,37,38]. Importantly, they found that theta and gamma rhythms can be used as markers for the development of native-language phoneme perception across the so-called sensitive period, a period that is characterized by infants’ initial ability, at 6 months, to perceive phonetic contrasts used to differentiate words across all languages, and then, at 12 months, to show a narrowing of their speech perception abilities as they begin to focus on phonetic units in the language(s) to which they are exposed [33,34,35,36,37,38].

Little is known about oscillatory networks underlying auditory temporal sampling in infants at risk for dyslexia in the first year of life, particularly across the sensitive period of native-language phoneme learning [11,37,38]. Some insight is offered by research on infants at risk for specific language impairment (SLI; suggested to share some genetic etiology with dyslexia) who showed atypical right-hemispheric theta and gamma processing in response to rapidly presented sounds at 6 months and its link to later expressive vocabulary [39]. These results suggest that infants at risk for dyslexia may already have an altered oscillatory response before they enter the critical period of native language learning, and it remains to be seen whether this response will change over the course of the sensitive period of native language learning. It is important to investigate oscillatory networks during this period because they could alter infants’ ability to process language(s) to which they are exposed. This, in turn, could have many consequences for later skills, such as the formation of precise phoneme perception, language, and reading—all of them reported to be symptomatic in dyslexia.

The present cross-sectional study examined auditory temporal processing in 6- and 12-month-old infants at risk for dyslexia and their age-matched controls. All infants came from monolingual English-speaking households, and thus, language as a possible confounding variable was kept constant. In contrast to prior EEG and NIRS research methods that provided only limited information on the spatial characteristics of neural activation [29,30,31,32,39], we employed MEG, a brain-imaging method with precise spatiotemporal resolution that records changes in the magnetic fields arising from neuronal activity [40]. We recorded auditory steady-state responses (ASSRs) to amplitude-modulated (AM) white noise, for which the AM rate linearly increased from 2 to 80 Hz, thereby covering the syllabic and phonemic sampling domain. ASSRs have the advantage that the modulating frequency of the sounds will be reflected in the neural response, and multiple modulation frequencies can be used in a single auditory stimulus to record ASSRs simultaneously [41]. This contrasts previous infant work that presented unmodulated tones and analyses focused on one or two frequency bands [31,32,39].

Our research is the first to investigate oscillatory responses of cortical neurons entrained by white noise modulated at frequencies relevant for speech processing in infants at risk for dyslexia and their matching controls. Because previous theories assigned theta a fundamental role in speech processing [7,11,42] and theta rhythms were found to be deficient in infants at risk for SLI [39], we hypothesized to find atypical neural locking at theta in response to the AM white noise in infants at risk for dyslexia. Moreover, because gamma rhythms were linked to phonological processing deficits in dyslexia [20,22], we also hypothesized to find atypical locking at gamma in at-risk infants. Finally, consistent with our position that auditory sampling during this sensitive period is critical to language learning and our prior work showing that infants’ early neural responses to simple sounds and language can predict later language [43,44], we further hypothesized that atypical auditory sampling at frequencies relevant for speech processing in at-risk infants would predict functional outcomes of later language skills. To test this, we correlated stimulus locking at frequencies relevant for language processing with later nonlinguistic communication and perceptive, expressive, and syntactic language skills at 13–30 months of age because similar measures were found to predict later literacy skills [45,46].

We found deficient theta locking in at-risk infants at both 6 and 12 months, indicating atypical auditory sampling at the syllabic rate in at-risk infants across the sensitive period for native-language phoneme learning. This interpretation is supported by our additional finding that auditory sampling at theta predicted later vocabulary comprehension, nonlinguistic communication, and the ability to combine words. Our results indicate a possible early marker of risk for dyslexia.

## 2. Materials and Methods

### 2.1. Participants

In this cross-sectional study, 31 6-month-old and 48 12-month-old infants participated, except for three infants that took part at both ages. All infants were English-learning, with English as the only language spoken at home. Infants were assigned to the control group when both biological parents had no prior diagnosis of dyslexia or reading problems and no biological relative with dyslexia or reading problems. Infants were assigned to the at-risk group when the biological parent with dyslexia had a prior diagnosis of dyslexia by a registered professional and a biological first-degree family member with a prior diagnosis of dyslexia or reading problems. Infants of biological parents with a history of other learning difficulties (e.g., attention deficit hyperactivity disorder, dyscalculia) or any type of language, speech, hearing difficulty or neurological disorder were excluded during recruitment. In addition, infants were excluded from this study if either biological parent or the biological parent with dyslexia had any known history of brain injury, a somatic or psychiatric condition affecting cognitive functions (including major depression), substance abuse, or medication affecting cognitive functions. Further, data from a total of seven 6-month-old and 17 12-month-old infants were excluded because of the inability to tolerate the head position indicator coils (9), the inability to localize the head position indicator coils in the MEG (6), a lack of a sufficient amount of 60 epochs during data collection (6), or a lack of a reliable dipole signal (3).

The final data set included 24 6-month-old and 29 12-month-old infants, with a 6-month-old group of 12 at-risk (5 males) and 12 control infants (5 males) and a 12-month-old group of 14 at-risk (6 males) and 15 control infants (7 males). All infants were born full-term (between 39 and 42 weeks of gestational age), had typical birth weight between 6 and 10 lbs with no major birth or postnatal complications, had no reported hearing difficulties, and had no history of recurrent ear infections. Mean age and gender were not significantly different between at-risk and control infants (mean age: one-way analysis of variance (ANOVA): *p* = 0.135 for 6-month-old infants (at risk: 190.83 ± 4.9 days, control: 193.75 ± 4.3 days); *p* = 0.457 for 12-month-old infants (at-risk: 373.1 ± 8.9 days, control: 370.6 ± 8.7 days), gender: Pearson’s chi-square: 6 months: *χ*^2^(1) = 0.0, *p* = 1.0, 41.7% males; 12 months: *χ*^2^(1) = 0.042, *p* = 0.837, 44.8% males).

### 2.2. Cognitive Testing

Parents’ Full-Scale IQ-2 (FSIQ-2) and Verbal IQ (VCI) were assessed with subtests for vocabulary, matrix reasoning, and similarity of the Wechsler Abbreviated Scale of Intelligence—Second Edition (WASI-II) [47]. Reading abilities were measured with subtests letter-word identification (LW), passage comprehension (PC), and word attack (WA), and spelling ability with Woodcock Johnson^®^ IV (WJ IV) Tests of Achievement Form A [48]. LW and WA were timed to evaluate the speed of single-word and pseudoword reading. Long-term retrieval was measured with subtests story recall and visual-auditory learning of the WJ IV Tests of Cognitive Abilities [49]. In this study, 62 out of 80 parents participated in this part.

### 2.3. Socioeconimic Status (SES)

Groups were matched on SES using the Hollingshead scale index [50] (46 out of 53 families completed the forms). Two-way ANOVA yielded no significant differences in SES between groups (*p* = 0.7) and ages (*p* = 0.169) (families of 6-month-olds: M = 52.95, SD = 9.6 (at-risk), M = 52.6, SD = 5.7 (control); families of 12-month-olds: M = 55, SD = 10.8 (at-risk), M = 57.3, SD = 6.4 (control)).

### 2.4. Stimuli

**Figure 1:**
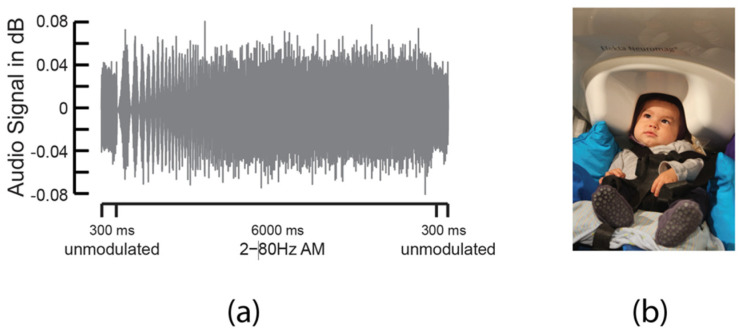
(**a**) Acoustic waveform showing 6000-ms-long 2–80 Hz AM white noise framed by 300 ms long unmodulated white noise. (**b**) Example of infant under the MEG helmet during recording.

ASSRs were measured using a 6000-ms-long AM white noise with its modulation rate linearly increasing from 2 to 80 Hz (Figure 1a) (stimuli adapted from Lehongre and colleagues [22]). This AM white noise was preceded and followed by 300 ms long white noise (total stimulus length: 6.6 s) with a constant envelope and a randomized white-noise carrier to diminish habituation effects of the signal throughout the experiment. Stimuli were presented with a silent interstimulus interval varying between 1 and 1.2 s at 65 dB SPL through loudspeakers using a Tucker-Davis Technology RZ6 real-time processor controlled by custom experimental software written in Python.

### 2.5. MEG Recording

MEG data were acquired inside a magnetically shielded room with a whole-head, adult-sized 306 channel Elekta Neuromag^®^ MEG system (Elekta Oy, Helsinki, Finland). Three anatomical landmarks (left and right preauricular points, nasion), five HPI coils, and approximately 100 additional points were digitized along the head surface using Fastrak^®^ 3D digitizer (Polhemus, Colchester, VT, USA) in order to construct an individual Cartesian head-centric coordinate system. Once the infant was seated calmly in a custom-made chair under the MEG helmet (Figure 1b), MEG data recording began with an analog band-pass filter of 0.03–330 Hz and a sampling rate of 1.2 kHz. During recording, infants were entertained with silent toys while a silent video of baby faces was played in the background. Infants’ head positions in relation to the sensor array were tracked continuously by extracting the magnetic fields emitted by HPI coils at frequencies between 83 and 323 Hz. Any channels with amplitudes lower than a certain level were marked as “flat”, removed (grad = 1 × 10^−13^; mag = 1 × 10^−15^), and reconstructed by applying the signal space separation (SSS) method [51] during preprocessing.

### 2.6. MEG Analysis

Using MNE-Python (version 0.19.2) [52,53], MEG data were processed using Maxwell Filter [53] to apply temporal SSS (tSSS) [54]. After tSSS, movement compensation [55] was applied (MaxFilter™ software, Elekta Neuromag^®^, Elekta Oy, Helsinki, Finland) and transformed to the median of each individual’s head position to minimize reconstruction noise. tSSS was performed in an 8-s time window with a correlation limit of 0.95 to reduce environmental noise. Further, automatic cardiac suppression with signal space projection using two magnetometer and two gradiometer projectors was applied.

Sensor-space MEG data were transformed to source-space data using a surrogate MRI from a 14-month subject segmented using FreeSurfer [56]. For each subject, digitizer data were used to rescale the 14-month MRI data to the subject’s individual head shape, and correspondingly rescale the inner skull and cortical surface meshes. There are four reasons we have confidence in using the 14-month-old template: (1) we previously successfully used the 14-month template brain in studies including those with 11-month-olds [57] 9-month-olds [58], and 7-month-olds [59]. (2) The differences (between 6 and 12 months) in scaling factors used to deform the surrogate MRI are relatively small. (3) The effects we find using the surrogate model are reflected in the individual ECD modeling, which does not use the surrogate model. Finally, (4) if there is some bias introduced, it should manifest as a main effect of age rather than as a main effect of group or an interaction term in the comparisons, so it is unlikely that this has affected our primary results.

After the rescaling process, the inner skull surface was used to compute a boundary element model (BEM) conductor model, and decimated cortical surfaces (4098 vertices per hemisphere) were used to construct a source space. A forward solution was then calculated using the linear collocation approach [40]. A sensor noise covariance was estimated from the baseline period (−0.2, 0 s) before each stimulus presentation, and combined with the forward to compute a minimum-norm inverse operator. To compute the minimum-norm solution, the regularization, depth weighting coefficient parameters were the default (1/9 and 0.8, respectively), dynamical statistical parameter mapping (dSPM) [56] noise normalization was used, and free orientation sources were assumed because of the use of a surrogate head model.

Stimulus AM envelope, sensor-space, and source-space data were each transformed into time–frequency responses (TFRs) using a continuous wavelet transform consisting of Morlet kernels from 2 to 80 Hz with 0.5 Hz spacing, each with a fixed duration of 1 s and a 40-ms time lag between the stimulus and brain response [60]. This fixed duration formed a time–frequency grid with uniform spacing across time and frequencies, designed to be consistent with methodology of previous studies [22]. Each frequency band in the TFR was then noise-normalized for each sensor and source point independently by subtracting the mean baseline level and dividing by the baseline standard deviation (z-scoring). Correlations with the stimulus were then performed for each sensor and source-space point by cropping the TFR to six different frequency bands (theta (4–7 Hz), alpha (8–12 Hz), low beta (13–25 Hz), high beta (25–35 Hz), low gamma (35–50 Hz), and high gamma (50–80 Hz)). To be conservative, we did not include the 2–3 Hz interval as it is likely too short to reliably capture locking to such low frequencies; 2/80ths of a linear frequency sweep that lasts 6 s will occur in just 150 ms, but a single full cycle of 3 Hz requires 333 ms. In sensor space, the variance explained for each hemisphere was computed by orthonormalizing (using coefficient of multiple correlation) the cropped TFRs for all sensors in a given hemisphere and computing the variance explained by the cropped stimulus AM TFR. The same procedure was used in source space for each source-space dipole by orthonormalizing the cropped TFRs for all three orientation time courses per source vertex. Correlation coefficients (*r^2^*) were obtained by correlating ASSRs with the stimulus AM for the left (L) and right (R) hemisphere sensors. Linear mixed-effects (LME) modelling as implemented in statsmodels [61] was used to compare at-risk and control infants with each frequency band (4–7 Hz, 8–12 Hz, 13–25 Hz, 25–35 Hz, 35–50 Hz, and 50–80 Hz), age (6 months; 12 months), and hemisphere (L; R) as separate factors (within-subject factors) according to the formula “corr ~ freq:group + freq + hemi + age”. Bonferroni correction was applied to the comparison of interest (interaction term between group and frequency) for the number of frequency bands.

### 2.7. Language Abilities

Language abilities were assessed with the MacArthur-Bates Communicative Development Inventories (CDI) [62]—a well-validated parent report measure. Comprehension and nonlinguistic communicative development were assessed in the same children at 13 and 15 months with subscales words understood, words produced, early and late gestures, and the sum of gestures of the infant form of the CDI. Expressive and syntactic language skills were evaluated at 18, 21, 24, 27, and 30 months with subsections words produced, irregular words, grammatical complexity, and M3L of the toddler form of the CDI. Parents typically completed CDI forms on the day their child reached the target age. The Pearson product-moment correlation coefficient was used to examine a possible association between the ASSRs and nonverbal and language skills measured with the CDI. ASSRs were collapsed across ages to increase statistical power.

## 3. Results

### 3.1. Cognitive Testing

Parents with dyslexia did not significantly differ from control parents in age, gender, and FSIQ-2, but showed significantly poorer, yet above average, VCIs and were significantly different from control parents in reading (LW; PC), basic reading (LW; WA), reading speed for words and pseudowords, spelling, and long-term retrieval (Table 1).

### 3.2. ASSR Reliability

**Figure 2:**
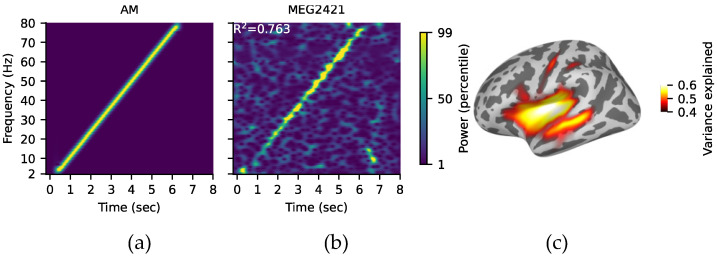
(**a**) TFR map of 2–80 Hz AM white noise. Warm colors indicate increase of oscillatory power. (**b**) TFR map of an example subject + sensor (MEG2421; a right temporal sensor). (**c**) Surface map for GA 3–5 Hz band correlation confirming maximal locking of the AM stimulus in the auditory cortices.

AM white noise ranging from 2 to 80 Hz evoked reliable ASSRs with high signal-to-noise ratio in all infants participating in this study. Figure 2 shows time-frequency representation (TFR) map of AM white noise (A), TFR map of an example subject + sensor (B) and surface map for grand-average (GA) 3–5 Hz band correlation confirming maximal locking of the AM stimulus in the auditory cortices (C). ASSRs were analyzed at theta (4–7 Hz), alpha (8–12 Hz), low beta (13–25 Hz), high beta (25–35 Hz), low gamma (35–50 Hz), and high gamma (50–80 Hz) to investigate potential differences in the locking of the stimulus AM in the auditory cortices between control and at-risk infants at 6 and 12 months.

### 3.3. LME Modelling

**Figure 3:**
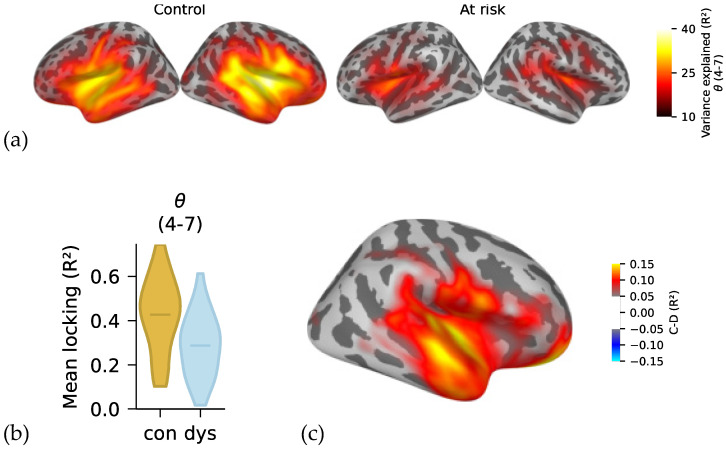
(**a**) GA maps in source space for theta (4–7 Hz) in control and at-risk infants collapsed across 6 and 12 months. (**b**) Violin plots show larger stimulus locking at theta for control than for at-risk infants (*p* < 0.001). (**c**) Group effect plotted on the brain surface (control infants minus at-risk infants).

Results of LME modelling are shown in Table 2. Groups were significantly different at the theta band (4–7 Hz), with larger locking for control infants than for at-risk infants over the entire brain surface (*r* = −0.14, *n* = 53, *p* < 0.001) (Figure 3). Results for the groups at other bands were nonsignificant after Bonferroni correction (*p* = 0.014–0.633).

### 3.4. Correlations with Later Language Skills

**Figure 4:**
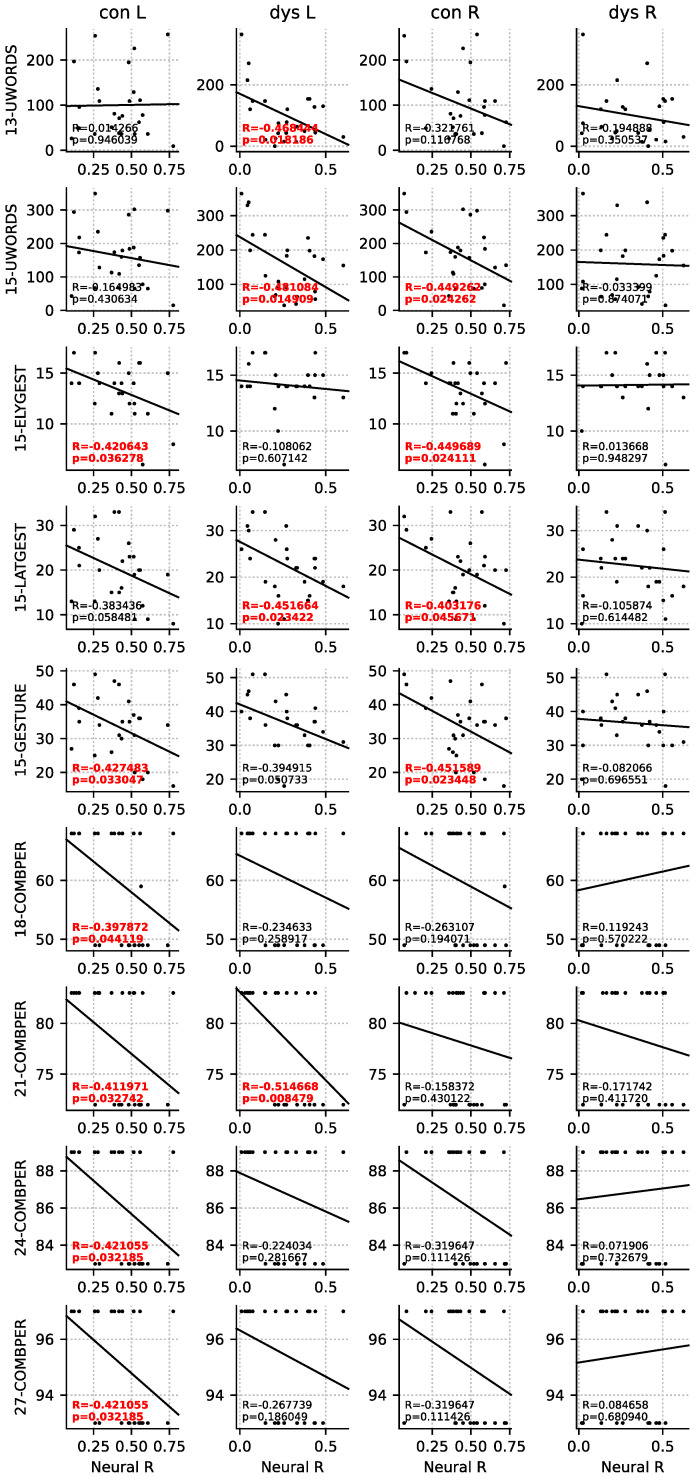
Scatterplots show significant correlations *(p* < 0.05, *r*- and *p*-values marked in red) between infants’ stimulus locking at theta (4–7 Hz) for the left and right hemisphere (L and R) and their language abilities at 13, 15, 18, 21, 24, and 27 months. Stronger left theta locking in at-risk infants predicted a lower number of words understood at 13 and 15 months. A similar pattern of response was observed in the right hemisphere for control infants. In addition, stronger left and right theta locking in control infants predicted the number of (early and late) gestures at 15 months, with a similar effect observed in at-risk infants for left theta locking and late gestures at 15 months. In addition, stronger left theta locking in control infants was linked to later lower percentile of combined words at 18, 21, 24, and 27 months, with the same correlation pattern found in at-risk infants at 21 months, but not at other measurement points.

ASSR-CDI correlation analysis was performed separately for the left and right hemispheres due to theoretical considerations of hemispheric differences between typical readers and readers with dyslexia for processing low-frequency temporal features in acoustical signals [42]. Analysis yielded significant negative correlations between theta locking and comprehension (words understood) and nonlinguistic communication skills (early and late gestures) at 13 and 15 months. Left theta locking predicted the number of words understood in infants at risk for dyslexia at 13 (Pearson *r* = −0.468, *n* = 25, *p* = 0.018) and 15 months (*r* = −0.481, *n* = 25, *p* = 0.015), whereas right theta locking predicted the same measure in control infants at 15 months (*r* = −0.449, *n* = 25, *p* = 0.024) (Figure 4). In both infant groups, stronger locking was linked to lower number of words understood. In addition, we found that left and right theta locking in control infants predicted the number of gestures at 15 months (left: *r* = −0.427, *n* = 25, *p* = 0.033; right: *r* = −0.452, *n* = 25, *p* = 0.023), with effects for both early (left: *r* = −0.421, *n* = 25, *p* = 0.036; right: *r* = −0.449, *n* = 25, *p* = 0.024) and late gestures (right: *r* = −0.403, *n* = 25, *p* = 0.046). This result was less consistent for infants at risk for dyslexia, with left theta locking solely linked to late gestures (*r* = −0.452, *n* = 25, *p* = 0.023). In both infant groups, stronger locking was linked to lower number of gestures.

Correlation analysis of left and right theta locking in at-risk and control infants with expressive and syntactic language skills at 18, 21, 24, 27, and 30 months yielded a significant link between left theta locking in control infants and later percentile of combined words at 18 months (*r* = −0.398, *n* = 26, *p* = 0.044), 21 months (*r* = −0.412, *n* = 27, *p* = 0.033), 24 months (*r* = −0.421, *n* = 26, *p* = 0.032), and 27 months (*r* = −0.421, *n* = 26, *p* = 0.032). Our analysis yielded the same correlation pattern for at-risk infants at 21 months (*r* = −0.515, *n* = 25, *p* = 0.008), but not for the other measurement points. The direction of this effect was the same for both infant groups, with stronger locking at theta predicting a lower percentile of combined words.

## 4. Discussion

This MEG study is the first to examine auditory cortical oscillations at frequencies relevant for speech analysis in infants at risk for dyslexia across the critical period for native phoneme learning. By recording ASSRs to non-speech stimuli that were amplitude-modulated at 2 to 80 Hz, we were able to test the functional integrity of auditory pathways and their ability to synchronize neural activity at several processing rates relevant for language. Our results demonstrate atypical auditory oscillatory sampling at theta in infants at risk for dyslexia across the sensitive period of native phoneme learning and its correlation to later language skills.

### 4.1. Auditory Sampling at Theta in TD Infants and at-Risk Infants

Typical sampling at theta was reflected in stronger stimulus locking for control than for at-risk infants at 6 and 12 months. This result is not surprising given that the integrity of auditory functioning at theta plays an essential role in the perceptional analysis of speech [7,8] and infants at that age enter a critical period of sound development by trying to master sounds of their native language(s) [35]. The phase pattern of theta was found to track and discriminate spoken sentences and to segment them into syllable-sized packages—a failure of which results in compromised intelligibility of speech [63,64]. Research on theta oscillations in TD infants is scarce, but we know that neural entrainment at theta is intact from birth [30] and marks several aspects of auditory and language development, such as discriminating nonverbal stimulus contrasts during rapid-rate presentation [31,32], neural commitment to native phoneme sounds [33,34], and processing of naturally sung nursery rhymes [65]. Our study adds that a typical response pattern of theta is reflected in enhanced neural entrainment (at least to white noise amplitude-modulated at syllable rate) in TD infants across this critical developmental period. This result is consistent with prior research showing that temporal processing at theta was enhanced in 6- to 15-month-old infants relative to adults for nursery rhymes [65]. We argue that enhanced theta entrainment likely promotes native phoneme learning as infants construct cortical maps for native language perception and processing during this critical time period [35,36,37,38].

Risk for dyslexia manifests itself in atypical auditory sampling at theta at 6 and 12 months, as reflected in decreased stimulus locking at theta in at-risk compared to control infants. This result is consistent with our hypothesis and supports child and adult data showing that risk for dyslexia is been associated with impaired auditory sampling at theta [13,14,15,16,17,18,19,20,23]. At a neurophysiological level, the strength of ASSRs depends on accurate phase-locking of neuronal populations to the temporal envelope of the stimulus [24]. Decreased strength of ASSRs at theta in at-risk infants could therefore result from a reduced number of neurons that phase-lock to temporal modulations, or alternatively, from asynchronous neuronal firing within a neuronal generator. Therefore, it is reasonable to assume that our results of decreased theta ASSRs in at-risk infants compared to control infants reflect a general neurophysiological abnormality in processing speech envelope cues at theta rate in these infants. Another possible explanation for impaired theta entrainment could be that sensory processing of acoustic cues that are required to entrain is impaired at a different level(s) of the auditory system, i.e., the number of neurons are there but they are not functioning as effectively. Support for this explanation comes from studies showing impaired discrimination of amplitude envelope rise time—a key sensory trigger for cortical tracking of the speech envelope—and its link to delayed language milestones (e.g., achieving phonological constancy) in the same infants in toddlerhood [66,67].

We found no effects of hemisphere for theta locking in both control and at-risk infants, a result that is different from adult data showing stronger theta locking in the right hemisphere in fluent readers and absent right-lateralized processing of theta oscillations in adults with dyslexia [6,20,23,68]. In contrast to adults, lateralization of theta oscillations is still emerging in infants, as demonstrated by some right-hemispheric preferences for processing of simple tones presented at theta rate in newborn to 6-month-old TD infants [30], but no hemispheric differences for processing nursery rhymes in 6 to 15-month-old TD infants [66]. One explanation for differences in results may be the type of stimuli used. Lateralization patterns for natural speech (such as nursery rhymes) or stimuli resembling the temporal pattern of speech (such as AM sounds used here) could emerge more slowly compared to lateralization patterns for simpler sounds in infancy. Moreover, infants master many developmental milestones in their first year, including native phoneme learning, for which they begin to construct cortical maps for native language perception and processing [35,36,37,38]. Because this is an ongoing learning process during the second half of the first year, lateralization may emerge later.

### 4.2. Gamma Sampling

Inconsistent with our hypotheses and prior findings in child and adult data [21,22], we found no significant differences between the groups for auditory sampling at gamma. This is unexpected because fast-spiking neurons in gamma oscillations make this band suitable for processing acoustic information varying in the range of tens of milliseconds, including the processing and discrimination of segmental sublexical information in phonemes—one key symptom in individuals with dyslexia [7,69,70]. One explanation for this lack of a group difference at gamma could be that oscillatory networks underlying the processing of spectrotemporal features in the auditory cortex refine with age. A recent longitudinal EEG study examined cortical entrainment of nursery rhymes (sung infant-directed speech) in TD infants aged 4, 7, and 11 months and reported both delta- and theta-band entrainment to speech already at 4 months of age, with the power of delta-band entrainment particularly strong at 4 months and theta-band power increasing over the first year of life [71]. Accordingly, it is likely that gamma-band entrainment develops across this sensitive period of native phoneme learning and effects can be observed later. An alternative explanation could be that gamma band processing is deficient in infants with dyslexia; however, because of analysis or stimuli used in the current study, the effect is not significant. Future research is needed to investigate this further.

### 4.3. Auditory Sampling at Theta and Its Correlation to Later Language Skills

Our results of stronger left theta locking and its link to lower number of words understood at 13 and 15 months are unexpected and contrary to our brain data that indicate that enhanced theta locking is typical during this sensitive period of language learning. When examining the 6- and 12-month-old data separately for each group, we found that this effect likely stems from the 6-month-old infants in the control group, *r* = −0.566, *n* = 11, *p* = 0.069; 12-month-old, *p* = 0.44 (15 months), and from the 12-month-old infants in the at-risk group, *r* = −0.473, *n* = 14, *p* = 0.087; 6-month-old, *p* = 0.133 (13 months), *r* = −0.57, *n* = 14, *p* = 0.033; 6-month-old, *p* = 0.218 (15 months). It is difficult to speculate about the exact cause of these results, but we know that infants’ brain mechanisms mature across the sensitive period for native phoneme learning, such as TD infants’ brains become more efficient in processing simple white noise between 6 and 12 months [44]. Although this is speculative, our ASSR-CDI results suggest that such efficiency processing seems to happen later in infants at risk for dyslexia compared to their age-matched controls. Interestingly, significant correlations were located over the left hemisphere in at-risk infants, whereas over the right hemisphere in control infants. This finding is consistent with research suggesting that the coding of the speech envelope is lateralized in the right hemisphere [72]; a function that is deficient in poor readers [11,12].

Our second result of stronger locking at theta and its correlation to lower number of gestures in control and at-risk infants at 15 months was equally unexpected. When examining the 6- and 12-month-old data separately for each group, we again found that control infants were significant in their ASSR-CDI correlations at 6 months (*r* = −0.566–−0.8, *n* = 11, *p* = 0.003–0.07) but not at 12 months (*p* = 0.347–0.848), whereas at-risk infants showed the reverse (12 months: *r* = −0.494, *n* = 14, *p* = 0.073; 6 months: *p* = 0.212). Therefore, our data suggest that infants at risk for dyslexia are sluggish in their efficiency processing relative to their age-matched controls. Our data show that significances in ASSR-CDI correlations for gestures were more consistent in control infants (six out of twelve correlations reached significance), whereas they were less pronounced for at-risk infants, with only one out of twelve correlations reaching significance. Given that the average number of gestures was similar for both groups (control: 33.44; at-risk: 36.64), it is likely that lower, and thus deficient, theta locking is the reason for this result.

Our third result of stronger locking at theta and its link to lower percentile of combined words at 18–27 months is interesting because our data showed that it stemmed from the 12-month-old data (*r* = −0.464–−0.536, *n* = 14–15, *p* = 0.048–0.12) and not from the 6-month-old data (*p* = 0.12–0.19) in both groups. This is reasonable given that combining words is a more advanced language ability compared to comprehension or nonlinguistic communication [73]. When observing the directions of the ASSR-CDI correlation in Figure 4, our data indicate that at-risk infants mostly rely on the left hemisphere, whereas control infants display a similar, although not significant, direction of the effect for the right hemisphere in addition to the left hemisphere. One may speculate that the groups are different in their hemispheric involvement for processing language already at that age.

### 4.4. Limitations

Phase synchronization at delta is viewed as a key acoustic statistic that supports the perception of speech rhythm, especially the extraction of the entire phonological hierarchy of syllable stress patterning, syllable boundaries, onset-rhymes, and phonemes [7,8,11,74,75]. To investigate this, we adapted stimuli from Lehongre and colleagues [22] by including slow delta amplitude modulations (2–4 Hz). However, our ability to actually measure the amplitude-modulation locking in the low delta frequency range was compromised because the stimuli did not include one full cycle or preferably multiple cycles to estimate frequency content. Specifically, the upper end of delta (4 Hz) requires 250 ms for one complete cycle, but our stimulus AM that ramped linearly from 2 Hz to 80 Hz over 6 s spent at best (4 − 2)/(80 − 2) = 2.5% of the time in the entire 2–4 Hz frequency range, which is ~150 ms and hence, much less than the time for one complete cycle. A future design should thus use a logarithmic rather than a linear sweep and/or longer durations to investigate lower frequency bands.

We chose to investigate auditory sampling in dyslexia with amplitude-modulated white noise because of its unique qualities, such that modulation frequencies used to elicit ASSRs are predefined and therefore, precise frequency analyses of responses can be performed at those frequencies [41]. However, it is possible that naturally produced continuous speech could facilitate successful cortical tracking of the speech stream in infants at risk for dyslexia, which, in turn, could assist those infants in the challenging task of learning their native language(s). For instance, recent research showed that infant-directed (IDS) compared to adult-directed speech facilitated cortical tracking of speech in TD infants [76]. Thus, future studies are needed to investigate the effects of IDS on oscillatory networks underlying speech processing in infants at risk for dyslexia.

Ideally, we would have preferred to adopt a longitudinal over a cross-sectional study design to examine differences between the 6- and 12-month-old cohorts, but this was not possible because of the complexity of successful MEG measurements, time constraints of the study, and limited number of at-risk children. Furthermore, future studies are needed to include follow-up measurements with the same children when they are at school age to investigate which children are diagnosed with dyslexia and whether decreased theta measures at this age could be a predictor of later symptoms of dyslexia.

## 5. Future Works

Our result of reduced low-frequency sampling in infants at risk for dyslexia brings us one step closer to defining core neural deficits underlying dyslexia, with the long-term goal to improve efficacy of interventions. Remediation would especially be important during the sensitive period when native phonemes are formed because we found that deficient theta locking across this period is linked to atypical language perception. Besides reading and language interventions for dyslexia that are commonly used with school-aged children [77], remediation practices based on rhythmic perception or music should be considered [78]. Practices could include moving infants in time with musical rhythms or rhythmic language (e.g., bouncing infants every time when a new syllable in a song or nursery rhyme occurs) or matching syllables and their patterns to metrical structure in music [11]. Because such practices can start at a very young age, they would have the potential benefit of improving auditory sampling in these infants during the critical time of native phoneme perception. This way, children can be equipped with tools to get them up to speed with their peers in reading.

## 6. Conclusions

Investigating oscillatory functioning underlying auditory sampling in dyslexia is an important aspect for early detection and for designing tailored interventions for children at risk for dyslexia before they enter school. Prior research in children and adults suggested that literacy problems in dyslexia can be traced back to atypical processing of auditory temporal modulations that are critical for accurate speech perception and production and subsequently for precise development of native phonological representations. Using MEG, the present study demonstrated that infants at risk for dyslexia show deficient processing of simple sounds modulated at the syllable rate, and that these difficulties relate to later language skills. Our results suggest that interventions based on rhythmic perception or music, such as bouncing infants every time when a new syllable in a song or nursery rhyme occurs, may be beneficial for addressing dyslexia at a very young age. This is especially critical during the sensitive period when native phonemes are formed. Moreover, deficient low-frequency phase locking likely has not only effects on the phonological development, but also implications for effective functioning of other cognitive tasks such as attention or memory, all processes that have been linked to slow oscillatory cortical functioning [1,2,3,4]. Hence, investigating how neural oscillations code important sensory parameters likely has implications for other learning difficulties beyond dyslexia.

## Figures and Tables

**Table 1:** Cognitive test results for parents of 6- and 12-month-old infants.

	Parent with Dyslexia	Control Parents	*F*	*p*	*η_p_* ^2^
Parents of 6-month-old infants (8 parents with dyslexia; 22 control parents)					
Age	33.75 (5) ^a^	32.86 (5.4)	0.164 ^b^	0.689	0.006
Sex ratio: male/female	5/3	11/11	*χ*^2^(1) = 0.368 ^c^	0.544	
FSIQ-2 ^d^	114.88 (13.7)	118.95 (12.4)	0.601 ^b^	0.445	0.021
VCI ^d^	109.88 (11.9)	119.77 (11.5)	4.270 ^b^ (4.564) ^e^	0.048 (0.042) ^e^	0.132 (0.145) ^e^
Reading ^d^	96.5 (7.7)	110.59 (10.2)	12.616 ^b^ (17.257)	0.001 (<0.001)	0.311 (0.390)
Basic Reading ^d^	93.5 (12.5)	110 (11.6)	11.384 ^b^ (14.484)	0.002 (0.001)	0.289 (0.349)
Reading speed (words)	169.3 (186.4) ^f^	86 (23.7)	4.459 ^b^ (3.654)	0.044 (0.067)	0.137 (0.119)
Reading speed (pseudo words)	116.98 (101.3) ^f^	71.59 (18.2)	4.296 ^b^ (3.506)	0.048 (0.072)	0.133 (0.115)
Spelling	45.4 (28.3) ^g^	70.5 (21) ^g^	6.998 ^b^ (6.298)	0.013 (0.018)	0.200 (0.189)
Long-term retrieval ^d^	107.4 (13.8)	117.59 (10.4)	4.755 ^b^ (3.931)	0.038 (0.058)	0.145 (0.127)
Parents of 12-month-old infants (10 parents with dyslexia; 22 control parents)					
Age	33.3 (5.7)	35.86 (3.4)	2.545 ^h^	0.121	0.078
Sex ratio: male/female	3/7	10/12	*χ*^2^(1) = 0.681	0.409	
FSIQ-2	112.3 (8.8)	120.5 (12.4)	3.594 ^h^	0.068	0.107
VCI	110.2 (9.7)	119 (10.8)	4.915 ^h^ (1.220)	0.034 (0.278)	0.141 (0.040)
Reading	99.3 (6.9)	111.2 (9.6)	12.383 ^h^ (7.783)	0.001 (0.009)	0.292 (0.212)
Basic reading	92 (9.1)	109.8 (10.5)	21.417 ^h^ (16.493)	<0.001 (<0.001)	0.417 (0.363)
Reading speed (words)	127.3 (37.9)	79.4 (21.2)	21.172 ^h^ (16.469)	<0.001 (<0.001)	0.414 (0.362)
Reading speed (pseudo words)	108.1 (21.2)	69.64 (19.1)	25.987 ^h^ (20.074)	<0.001 (<0.001)	0.464 (0.409)
Spelling	40.6 (17.9)	73.32 (16.06)	26.571 ^h^ (20.215)	<0.001 (<0.001)	0.470 (0.411)
Long-term retrieval	107 (14.8)	117.91 (11)	5.727 ^h^ (3.047)	0.023 (0.091)	0.160 (0.095)

^a^ Standard deviations are given in parentheses; ^b^ *F*(1,29); ^c^ Pearson’s chi-square test; ^d^ standard scores^; e^ ANCOVA, *F*- and *p*-values are reported after controlling for FSIQ2 in parentheses; ^f^ in seconds; ^g^ national percentile rank; ^h^ *F*(1,31).

**Table 2:** LME results for group difference (*n* = 53) at each frequency band.

					Confidence Interval	
	Coefficient Estimate	Standard Error	*z*	*p > |z|*	[0.025]	0.975]
Intercept	0.4	0.02	19.701	<0.001	0.36	0.439
Frequency Bands						
4–7 Hz	−0.14	0.026	−5.387	<0.001 *	−0.192	−0.089
8–12 Hz	−0.064	0.026	−2.46	0.014	−0.115	−0.013
13–25 Hz	−0.026	0.026	−1.002	0.316	−0.077	0.025
25–35 Hz	0.012	0.026	0.477	0.633	−0.039	0.064
35–50 Hz	−0.014	0.026	−0.539	0.590	−0.065	0.037
50–80 Hz	−0.013	0.026	−0.488	0.626	−0.064	0.038

* Significant after Bonferroni correction (0.05/6 = 0.0083); significant effect for theta band (4–7 Hz) is shown in Figure 3.

## Data Availability

All relevant data supporting the findings of this study are stored on a server at the Institute for Learning & Brain Sciences at the University of Washington and are available for research purposes on request by contacting P.K.K.
